# ncRNAclassifier: a tool for detection and classification of transposable element sequences in RNA hairpins

**DOI:** 10.1186/1471-2105-13-246

**Published:** 2012-09-25

**Authors:** Sébastien Tempel, Nicolas Pollet, Fariza Tahi

**Affiliations:** 1 Laboratoire IBISC-IBGBI EA 4526, Université d’Evry-Val d’Essonne, 91034 EVRY, Genopole, 23 Bd de France, France; 2 Metamorphosys iSSB, CNRS, Université d’Evry-Val d’Essonne, 91058 Evry, Genopole, Genavenir 3 - Genopole campus 1, 1 rue Pierre Fontaine, France

## Abstract

**Background:**

Inverted repeat genes encode precursor RNAs characterized by hairpin structures. These RNA hairpins are then metabolized by biosynthetic pathways to produce functional small RNAs. In eukaryotic genomes, short non-autonomous transposable elements can have similar size and hairpin structures as non-coding precursor RNAs. This resemblance leads to problems annotating small RNAs.

**Results:**

We mapped all microRNA precursors from miRBASE to several genomes and studied the repetition and dispersion of the corresponding loci. We then searched for repetitive elements overlapping these loci. We developed an automatic method called ncRNAclassifier to classify pre-ncRNAs according to their relationship with transposable elements (TEs). We showed that there is a correlation between the number of scattered occurrences of ncRNA precursor candidates and the presence of TEs. We applied ncRNAclassifier on six chordate genomes and report our findings. Among the 1,426 human and 721 mouse pre-miRNAs of miRBase, we identified 235 and 68 mis-annotated pre-miRNAs respectively corresponding completely to TEs.

**Conclusions:**

We provide a tool enabling the identification of repetitive elements in precursor ncRNA sequences. ncRNAclassifier is available at http://EvryRNA.ibisc.univ-evry.fr.

## Background

A central problem with small RNA transcriptomics is to identify degradation products and to sort small non-coding RNA sequences into functional categories. Functional small RNAs (miRNAs, snoRNAs, siRNAs ..) are produced by several biosynthetic pathways that metabolize hairpin structures formed by precursor RNAs originating from inverted repeat genes [1,2]. The occurence of such hairpins in large genomes is frequent, with 10^5^ to 10^6^ hairpins for a typical vertebrate genome. Most of these genomes are transcribed (93% for the human genome) [3] and then processed into large and small RNA pieces, including hairpin structures [4]. It turns out that a majority of these hairpins are components of transposable elements (TEs).

TEs are functional elements that can change their genomic location through either movement or duplication [5]. TE alone represents a substantial fraction of many eukaryotic genomes [6]. TEs are characterized and classified on the basis of terminal and/or sub-terminal structures and/or on their protein-coding capacity [7]. TEs are conventionally divided into two classes: Class I and Class II. Class I elements (retrotransposons) use reverse transcription from a RNA intermediate and Class II elements (DNA transposons) are characterized by terminal inverted repeats (TIRs) and are mobilized by a transposase [5]. Many TE families do not show any protein-coding capacity and are called non-autonomous transposable elements [5]. They accumulate so many mutations, insertions or deletions that they are generally defined only by their terminal repeats [8,9]. For example, Short INterspersed Elements (SINEs) like *Alu* are non-autonomous Class I elements characterized by short sequences (100–500 nt) that present stable secondary structures similar to the fusion of a tRNA and a hairpin structure [10,11]. Another example is provided by Miniature Inverted-repeat Transposable Elements (MITEs), non-autonomomous Class II elements characterised by a small size (80–500 nt) and a stable hairpin secondary structure [12].

**Figure 1:**
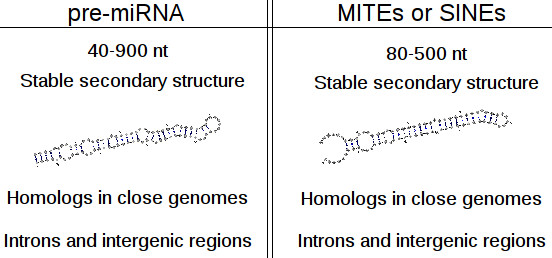
**non-autonomous TEs and inverted repeat genes share biological features.** The figure enumerates some characteristics that are shared by both non-autonomous TE and inverted repeat genes, such as hairpin structure. The structures given here correspond to CEL-LET-7 pre-miRNA in nematode and to an occurrence of MADE1 TE in human genome.

Short non-autonomous TEs and some non-coding precursor RNAs such as pre-miRNAs are characterized by a similar size and a hairpin secondary structure (Figure 1). Therefore, these two genetic entities can be defined as inverted repeat genes [4]. For example, the human MITE Hsmar1 sequence is 80 nt long and it forms a hairpin secondary structure [13]. Transcription of such MITEs by RNA polymerase II can lead to the synthesis of repeat associated small interfering RNAs (rasiRNAs) and to piwi RNAs. These small RNAs are similar in size to miRNAs [14-16]. Moreover, rasiRNAs trigger post-transcriptional regulations using DICER-like proteins just like miRNAs do [14,16].

Studies of Landgraf *et al.* and Piriyapongsa *et al.* describe miRNA genes originating from non-autonomous TEs [17-19] and recent studies claim that some pre-miRNAs share their sequences or an important part of their sequences with TEs [20-23]. Such cases of pre-miRNAs have been annotated in miRBase [24] and called TE-derived miRNAs [20].

The observation that some ncRNA sequences (miRNA among others) are similar to clearly identified TE sequences is reminiscent of old observations and problems. For example, the ubiquity of Alu repeats in human DNA has long been recognized as a problem for analysing human DNA and protein sequences [25]. It is therefore not surprising that small RNA sequencing surveys identify repeat and TE-derived small RNAs. Indeed, current bioinformatic pipelines designed for the analysis of small RNA sequences contain modules to identify reads that map to many genomic loci and discard them for further analysis [26]. For example miRDeep [27] discards reads that map to more than five positions in a genome, however this threshold is arbitrary and based on unpublished observations.

As always, such pipelines are limited as the vast number of TE-derived sequences results in the passage of some TE-derived small RNA sequences through the filter. This is due mainly to two things: 1) TE sequences in Repbase are represented by a single consensus sequence for a given TE family and 2) TE sequences are usually very polymorphic. Thus, small RNA sequences derived from TE are now represented in miRBase and users are in need of a tool to help them annotate small RNA sequences related to TEs. Moreover a relevant question for the evolutionary studies on small RNAs is whether this relationship between ncRNA and TE is a physiological process or a molecular background due to enzymatic promiscuity [28].

MicroRNAs (miRNAs) are small ncRNAs involved as regulators of gene expression at the post-transcriptional level by binding to specific target mRNAs whose translation are inhibited or down-regulated [29,30]. miRNA genes is transcribed and then cleaved into long precursors of miRNA [31]. These miRNA precursors (pre-miRNAs) are then cleaved into mature miRNAs of 21–25 nt length by Dicer proteins [31]. In the RISC complex, a mature miRNA binds with a specific mRNA transcript and leads to a cleavage/degradation, or a destabilization of the mRNA, both usually leading to downregulation of this mRNA [29,31].

Criteria to annotate microRNAs were proposed in 2003 and evolved to take into account the data produced using massively parallel sequencing technologies [24]. However, some studies show that some microRNA genes are mis-annotated. For example Yan *et al.* showed experimentally that OSA-MIR441 and OSA-MIR446 correspond to small interference RNAs [14]. Langenberger and colleagues showed that snoRNA were often mis-annotated as microRNA [28]. In another example, a microRNA gene is entirely included in a TE; this is the case of HSA-MIR-1255a present on chromosome 4 [24]. This locus corresponds also to the MITE Tigger1 (Additional file 1). The same situation is found for all 58 members of the HSA-MIR-548 family.

In this article, we look at small RNAs from the point of view of TEs and propose a classification tool to sort them according to their similarities to TE sequences. We present an automatic method called *ncRNAclassifier* for classifying ncRNA precursors into three categories based on the percentage of TE in their sequence and their dispersion in the genome: 

• precursors whose sequence is devoided of TE-derived sequences and not repeated nor dispersed to a significant extent in the genome: *bona fide* pre-ncRNAs (or ncRNA genes).

• precursors whose sequence corresponds to a small part of a known TE sequence and/or that are repeated and dispersed in the genome: TE-derived ncRNAs.

• precursors whose sequence corresponds to a large part of a known TE sequence; either already annotated as such or identified by our method: mis-annotated ncRNAs.

Using ncRNAclassifier, we analysed pre-miRNA sequences from several genomes: frog, human, mouse, nematode, rat and sea squirt from the miRBase database ( http://www.mirbase.org) [24]. We found that hundreds of human and mouse pre-miRNAs, and some frog, nematode, rat and sea squirt pre-miRNAs, can be classified as being derived from TEs. We also observed numerous examples of pre-miRNAs corresponding completely to TEs that should therefore be re-annotated as TEs.

## Results and discussion

### Methodology overview

The number and the distribution of inverted repeat gene occurrences in the genome is an important feature which we used to link TEs that can still transpose with ncRNA genes. For example, miRNA genes are not associated with a transposition mechanism and are not widespread [18,32]. However, the local duplication of ncRNA genes by unequal crossover can lead to clusters such as those described for miRNAs [33]. Still, this mechanism does not create many widespread copies, and the existence of such clusters is recognized when the distance between two inverted repeat genes is less than 20,000 nt [33]. Here, our definition is that two inverted repeat gene occurrences are not in the same cluster if they are on different chromosomes or are seperated by at least 100,000 nt. We postulated that a pre-miRNA having several occurrences and/or present in several chromosomes have a strong probability to be mis-annotated.

**Figure 2:**
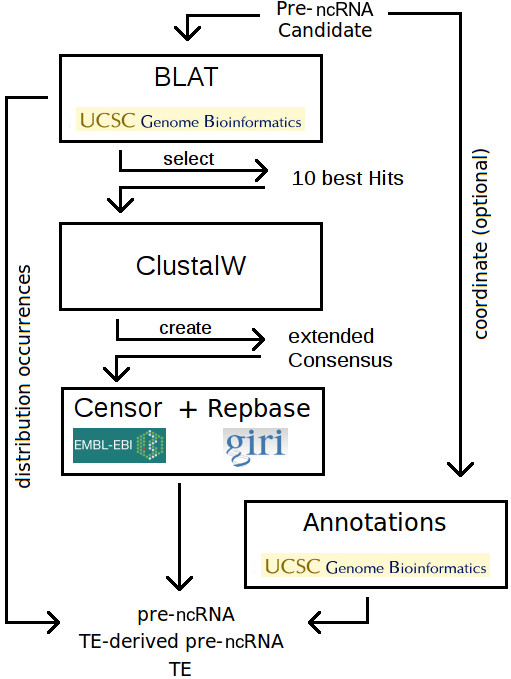
**ncRNAclassifier computational pipeline.** Giving a ncRNA candidate, the first step is to perform BLAT of the UCSC Genome Browser and to get the ten most similar hits. The second step is then to align these hits by ClustalW in order to get a consensus sequence that is extended. The last step is finally to match the extended consensus sequence with RepBase database using CENSOR of EBI.

**Figure 3:**
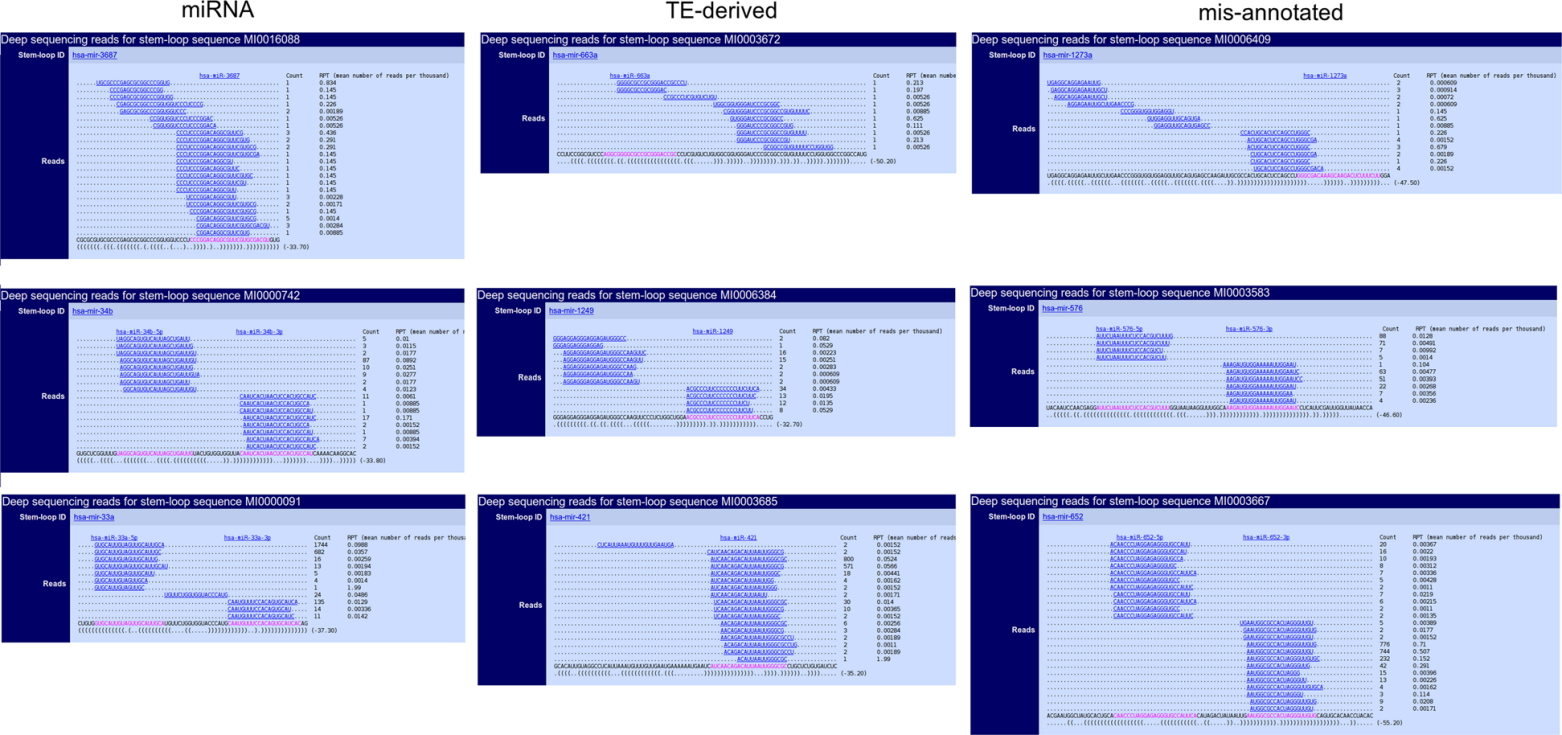
**Examples of deep sequencing of *bona fide*, TE-derived and mis-annotated pre-miRNAs from miRBase.** The figure presents nine examples of *bona fide*, TE-derived and mis-annotated pre-miRNA sequences. For each category, we present an example where the prominent mature ncRNA corresponds to about 30%, 60% and more than 90% of the total of mature ncRNA found by deep sequencing.

We present the overall workflow of ncRNAclassifier in Figure 2. In the first step of our method, we study the distribution of the occurrences of a query sequence using BLAT [34] at the UCSC Genome Browser [35]. BLAT returns sequence occurrences (“hits”) that are similar to the given precursor sequence, and the chromosomes where they appear. We chose BLAT at the UCSC Genome Browser because it refers to chromosomal location when this information is available, while BLAST at NCBI or EBI provide results as scaffolds location. A reference to scaffolds hinders the study of the occurrences because we cannot know if two occurrences appear in the same chromosome or in two different chromosomes. We then deduce the number of “similar hits”, which are hits whose similarity with the candidate is equal to or greater than 80% and whose size is between 80% and 120% of the precursor size. These thresholds are also used in [17]. Next, we calculate the number of chromosomes containing these similar hits. The number of similar hits and the associated number of chromosomal locations are important since *bona fide* pre-miRNAs are typically not found dispersed nor repeated in the genome. We found (Figure 3) that a candidate with at least 20 similar hits or present in more than six chromosomes/scaffolds is a TE-derived pre-miRNA or TE. We extract the ten best similar hits using UCSC genome browser [35] because this is enough to create a consensus sequence since the hits have a similarity with the precursor sequence greater than 80%.

In the second step, we fetch the surrounding sequence around each hit: 100 nt to the left and to the right. We need these additional bits of sequence because the size of some ncRNA precursors could be too short for the evaluation of possible similarities with known transposable elements. For example, human pre-miRNAs range between 60 and 140 nt [24]. The obtained sequences are then aligned using ClustalW [36] and a consensus sequence is created. The nucleotide consensus at position *i* corresponds to the most frequent nucleotide if it occured at least five times and ‘N’ otherwise.

In the third step, we use CENSOR [37] to compare the consensus sequence created previously to the RepBase TE database [13]. We chose CENSOR instead of Repet [38] because to our knowledge there is no Repet webserver. We preferred CENSOR to RepeatMasker (www.repeatmasker.org) because our method extracts the RepeatMasker annotation from UCSC genome browser [35] and CENSOR can show complementary results.

In the optional fourth step, activated when the user enters the ncRNA genomic coordinate, our method checks the RepeatMasker annotation from the UCSC genome browser [35]. The CENSOR results and the RepeatMasker results are then compared and the greatest TE fragment is kept.

The fifth step deals with the classification. We distinguish two cases. The first case is when a 24 nt segment (size of a mature mi- or siRNA [31]) unrelated to a TE sequence can be found. Thus, a mature small RNA could be generated from this precursor, and be able to bind to a target mRNA devoided of TE sequence. We call this a TE-derived pre-ncRNA. In the second case, no such segment can be found. Thus a mature small RNA generated from such a precursor would bind a target mRNA through a TE sequence. We call this a TE or a mis-annotated TE pre-ncRNA.

Since the interspersion of ncRNA precursor depends of the size and the number of chromosomes in a genome, the user can choose the thresholds that classify the ncRNA precursor (i.e., the minimal number of similar hits and the minimal number of chromosomes).

Finally, our method uses the occurrence distribution and the size of the recognizable TE sequence to classify the pre-ncRNA candidate. Based on these two features our method classifies the candidate according to the following rules: 

• one occurrence, no recognizable TE ⇒ *bona fide* pre-ncRNA

• more than 20 occurrences, no recognizable TE ⇒ TE-derived pre-ncRNA

• occurrences on six or more chromosomes, no recognizable TE ⇒ TE-derived pre-ncRNA

• one or more occurrences, segment unrelated to a TE ≥ 24 nt ⇒ TE-derived pre-ncRNA

• one or more occurrences, segment unrelated to a TE < 24 nt ⇒ TE

### ncRNAclassifier

**Figure 4:**
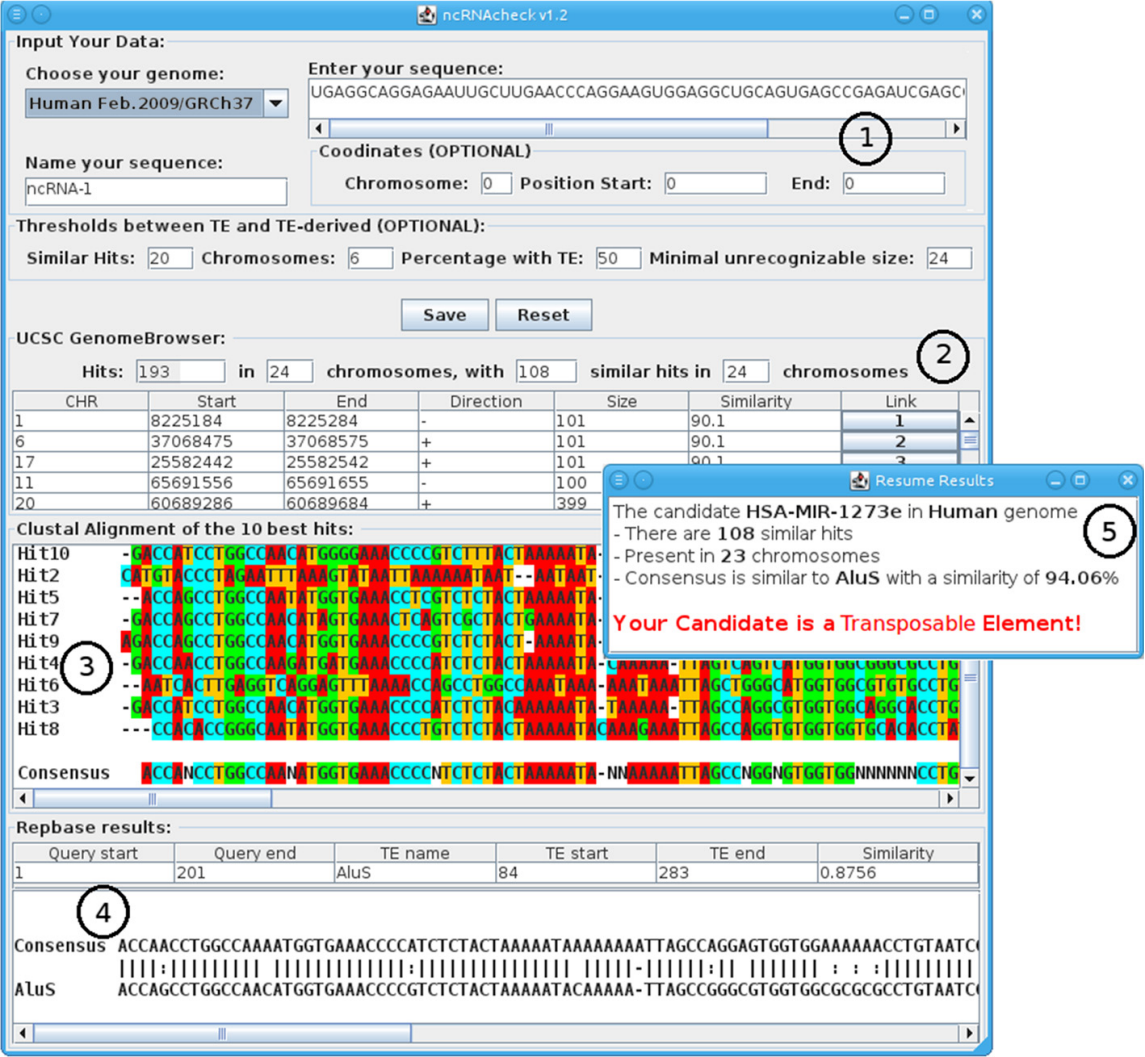
**JAVA interface of ncRNAclassifier.** It shows the results of the human miRNA HSA-MIR-1273e analysis and the intermediate results obtained at each step.

We call our method ncRNAclassifier (Figure 4) and a Java implementation is available at http://EvryRNA.ibisc.univ-evry.fr.

The interface of ncRNAclassifier works as follows: the user enters the sequence of a given pre-ncRNA candidate (for example a pre-miRNA) in STADEN format (1 in Figure 4), enters a name and chooses the corresponding genome. The hits found in the genome are displayed as a table (2 in Figure 4). The line above the table of hits summarises the BLAT results: (i) the number of hits returned by BLAT, (ii) the number of chromosomes where the hits appear, (iii) the number of similar hits (i.e., hits that have a size between 80% and 120% of the miRNA size and that have a similarity greater than 80% with the candidate sequence), and (iv) the number of chromosomes where similar hits appear. The user can check the hits obtained from BLAT using the link to the BLAT webpage storing the results: a pop-up window shows the BLAT alignment obtained by the UCSC genome browser. The extended hit sequences aligned by ClustalW and the consensus sequence generated are displayed (3 in Figure 4). The alignment between the consensus and the most similar TE is shown below (4 in Figure 4). If the user enters the coordinate of a pre-miRNA candidate (chromosome, position start and end (1 in Figure 4), ncRNAclassifier sends a request at the UCSC Genome Browser and gets the RepeatMasker annotation. This annotation is compared to CENSOR result and our method considers only the largest part of TE identified from them. Finally, a pop-up summarises the results and specifies if a given pre-ncRNA candidate corresponds to a TE or not (5 in Figure 4). After the ncRNAchek run, the ‘search’ button is replaced by a ‘reset’ and a ‘save’ button. The ‘reset’ button erases all data from the interface for a next run. The ‘save’ button saves the results into a text file. A multiple sequences analysis by ncRNAclassifier can be done by a command line with the ‘-g’ option.

Our interface does not use CENSOR or Blat directly but sends request to the EBI and UCSC websites where CENSOR or Blat are integrated. As these websites are frequently updated, they use the last version of these software. At the time of writing, RepeatMasker version was 3.3.0, CENSOR version was 4.2.27 and BLAT version was 3.4. RepeatMasker, CENSOR and Blat were used with their default parameters. The RepBase is also updated frequently on EBI. The last version of RepBase was 17.02 ( http://www.girinst.org/repbase/) [13] when we wrote this article.

### Analysis of pre-miRNAs from miRBase

We used ncRNAclassifier to analyze pre-miRNAs from miRBase [24] for six genomes: frog (*Xenopus tropicalis*), human (*Homo sapiens*), mouse (*Mus musculus*), nematode (*Caenorhabditis elegans*), rat (*Rattus norvegicus*) and sea squirt (*Ciona intestinalis*). The whole results are provided in Additional file 2.

We present our results concerning the number of TE-derived and the number of mis-annotated pre-miRNAs from miRBase for the six considered genomes in Table 1. In the human genome, TE-derived and mis-annotated pre-miRNAs represent 27.18% of all human pre-miRNAs in miRBase. In particular we observed a set of 11 human TE-derived pre-miRNAs composed of two or more TE fragments. For example, HSA-MIR-626 pre-miRNA is composed of two fragments of L1MB8 TE. Similarly, we observed that 48 human mis-annotated pre-miRNAs contain two distinct TE fragments, of which 15 are composed of two distinct families of TEs. For example, HSA-MIR-5095 is composed of a CHARLIE1A fragment (position 1 to 54) and a ALUSq2 fragment (position 55 to 89) and HSA-MIR-720 is composed of a HERVS71-int fragment (position 1 to 32) and a LTR6B fragment (position 34 to 109).

**Table 1:** Number of pre-miRNAs from miRBase that are TE-derived or mis-annotated

	**Total of pre-miRNAs**	**Mis-annotated**	**TE-derived**
Frog	182	1	3
Human	1037	235	152
Mouse	542	68	110
Nematode	200	2	5
Rat	359	28	21
Sea squirt	310	2	19

Number of pre-miRNAs from miRBase that are TE-derived or mis-annotated for frog, human, mouse, nematode, rat, and sea squirt genomes.

In the mouse genome, TE-derived and mis-annotated pre-miRNAs represent 24.72% of mouse pre-miRNAs in miRBase. We observed 16 pre-miRNAs (one mis-annotated and 15 TE-derived) containing two TE fragments. For example, the MMU-MIR-3471-1 miRNA contains MTA_MM and MusHAL1 TEs that are respectively an endogenous retrovirus and a L1 family. Like HSA-MIR-720 pre-miRNA, the two TE sequences contained in this mouse pre-miRNA are adjacent.

In the sea squirt genome, the proportion of pre-miRNAs identified as corresponding to TEs was 0.65%. One of the mis-annotated pre-miRNA corresponds completely to the HAT5N_CI transposable element. In the nematode genome, the proportion is of 1%. In the frog genome, there is one mis-annotated pre-miRNA and only three TE-derived pre-miRNAs. Finally, we found 28 mis-annotated and 21 that are TE-derived in the rat genome. We observed that eight rat mis-annotated pre-miRNAs correspond completely (at 100%) to TEs.

In conclusion, we found cases of mis-annotations and evident relationships with TE in the six genomes studied, with a positive correlation between the number of pre-miRNAs described in miRBase for a given genome and the number of pre-miRNAs related to TEs.

Among the 3276 pre-miRNAs that ncRNAclassifier classifies as mis-annotated or TE-derived, 267 pre-miRNAs present only RepeatMasker annotations (mainly SINE, L1 and CR1 transposons) and 104 pre-miRNAs present only CENSOR matches (mainly DNA transposons). Almost 11% of candidates show TE sequences with only method. This result shows the complementarity of CENSOR and Repeat Masker. ncRNAclassifier needs both methods to find the largest number of TE sequences. Among the 104 pre-miRNAs that present only CENSOR matches, 52 show TE sequences only when they are extended. For example, HSA-MIR-3176, HSA-MIR-3689c, MMU-MIR-551b and MMU-MIR-692-1, that are respectively TE-derived, mis-annotated, TE-derived and TE-derived miRNAs, show TE sequences inside precursors only if they are submitted with the extended sequence in CENSOR. These four examples have a similarity of about 70% with TE sequences. Because of these low similarities, it is difficult to find a “perfect seed” necessary for the BLAST-like algorithms (RepeatMasker and Censor). These results show that it is important to extend the ncRNA sequences to detect TE sequence with a low similarity.

### Deep sequencing data analysis of pre-miRNA categories defined by ncRNAclassifier

We extracted the data provided by miRBase for each human miRNA, counted the number of short reads starting at the same 5’ base, and computed a “predominance ratio” between the number of the most frequent short reads and the total number of short reads. This predominance ratio was used as an indicator of the biosynthesis of mature miRNAs but not as an indicator of miRNA expression level. Indeed, miRNA biosynthesis should lead to one or few overlapping predominant mature miRNA molecules coming from the cleavage of the pre-miRNA by DICER [29,31].

Firstly, we observed that about one third (35 to 40%) of the entries are lacking deep sequencing data in miRBase. The lack of sequencing reads for these pre-miRNAs might be explained by the lack of incorporation of some GEO data sets in miRBase. Moreover, for some miRNAs with special spatio-temporal pattern of expression, their corresponding libraries might have not yet been sequenced.

Secondly, we observed a similar predominance ratio profile for TE, TE-derived and *bona fide* miRNA categories: one third of a given category entries exhibit a predominance ratio of 90 to 100%. Thus, a single or few small RNA molecule species are produced from these hairpins. We conclude from these observations that deep sequencing data at a first glance can not distinguish TE and TE-derived from *bona fide* miRNAs. We used the *χ*^2^ statistical test to test for a difference between the distribution of prominent mature ncRNA in the three categories. A statistical difference would require a *χ*^2^ value higher than 16.919. When we compared the distribution of predominance ratio for miRNAs versus TEs and miRNAs versus TE-derived we obtained *χ*^2 ^values of 9.038 and 10.49, respectively. These two results show that deep sequencing data alone, without expert knowledge, cannot give the evidence that a sequence belongs to a miRNA and not a TE, and vice versa.

For example, HSA-MIR-1302-11 and HSA-MIR-1299 correspond completely to TEs and possess also mature ncRNAs. Moreover, Figure 3 shows the deep sequencing of nine precursors from miRBase. There are three precursors of each category. Figure 3 gives examples of *bona fide* pre-miRNAs that do not have prominent mature ncRNAs, and examples of mis-annotated sequences that have a prominent mature ncRNAs. On the other hand, the *bona fide* miRNA precursor HSA-MIR-103b-2 does not have known mature ncRNAs in miRBase and the *bona fide* miRNA precursor HSA-MIR-126 does not have a very clean Dicer cleavage indicative of the miRNA maturation.

Moreover, some predicted mature miRNAs are inconsistent with the ncRNAs obtained by the deep sequencing. For example, the miRNAs HSA-MIR-1234, HSA-MIR-1273a and HSA-MIR-5096 show a difference between mature miRNA and ncRNA described.

We finally decided to ignore the RNASEQ deep sequencing annotations found on miRBase since these data are unavailable for most ncRNA sequences. It is the case for instance of frog, nematode, rat and sea squirt genomes. Moreover, the number of deep sequencing reads is often insufficient to show a very clean Dicer cleavage indicative of a *bona fide* miRNA [29,31]. Additionally, the following results show that some *bona fide* miRNAs do not show with the analysis of RNASEQ deep sequencing the clean Dicer cleavage and some mis-annotated pre-miRNAs have this clean Dicer cleavage.

### Interspersion and distribution of pre-miRNA occurrences and their correspondence to TEs

**Figure 5:**
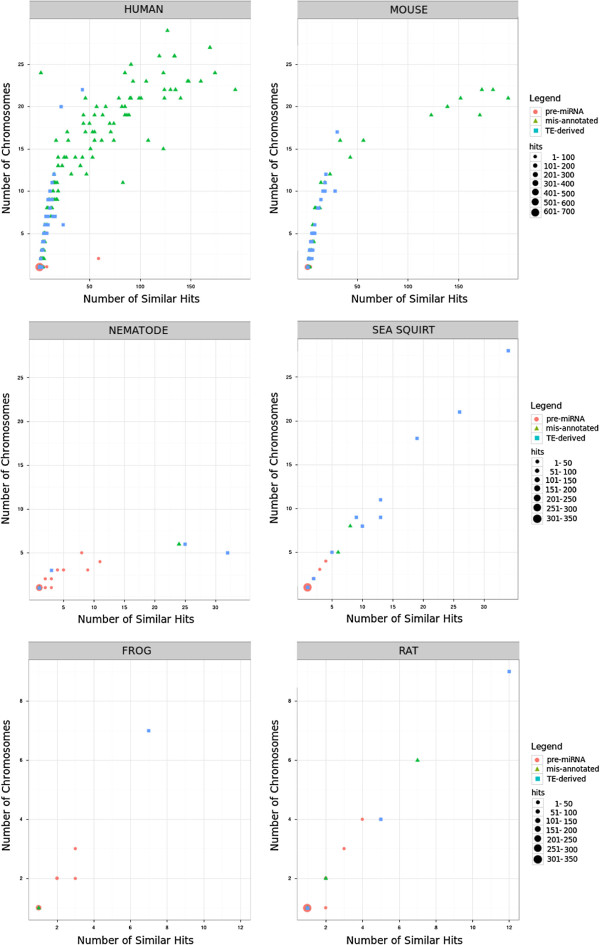
**Distribution of pre-miRNA hits in frog, human, mouse, nematode, rat and sea squirt genomes.** In red: pre-miRNAs identified by ncRNAclassifier as pre-miRNAs not corresponding to TEs. In blue: pre-miRNAs identified as TE-derived. In green: pre-miRNAs identified as TEs. The size of the dots depends on the number of considered pre-miRNAs.

We examined the interspersion and the distribution of pre-miRNA occurrences (Figure 5) according to the categories defined by ncRNAclassifier on a set of six genomes.

We found a positive correlation between mis-annotated or TE-derived precursor pre-miRNAs and the number of similar hits (Figure 5). Mis-annotated pre-miRNAs were characterized by the highest number of hits and the highest dispersion on chromosomes. TE-derived pre-miRNAs were characterized by fewer similar hits on less chromosomes than mis-annotated ones, and pre-miRNAs without TE-sequence have the lowest number of hits. This result is particularly remarkable on the human and mouse genomes.

We observed that a majority of pre-miRNAs that do not correspond to known TEs have only one similar hit (Figure 5). Only 36 pre-miRNAs among the total of 3,276 pre-miRNAs analysed in the six species (1.1%) have more than 20 similar hits or are present in more than 6 chromosomes but classified as unrelated to TEs by ncRNAclassifier.

Table 2 shows the number of pre-miRNAs that are identified as mis-annotated or TE-derived, according to their number of hits and their interspersion in the genome. Excepted for the rat, there is always more mis-annotated pre-miRNAs with many similar hits on one or several chromosomes than mis-annotated pre-miRNAs with a single similar hit. In all species, the number of TE-derived pre-miRNAs with only one similar hit is higher than the number of TE-derived pre-miRNAs with many hits on one chromosome. Excepted for the mouse, the number of TE-derived pre-miRNAs with only one similar hit is also higher than the ones with many hits on many chromosomes.

**Table 2:** Number of pre-miRNAs, TE-derived pre-miRNAs and mis-annotated pre-miRNAs in the six genomes

		**Frog**	**Human**	**Mouse**	**Nematode**	**Rat**	**Sea squirt**
1 similar hit	pre-miRNA	150	999	480	182	331	275
and	TE-derived	0	101	50	2	19	8
1 chromosome	mis-annotated	1	101	35	1	26	0
several similar hits	pre-miRNA	0	47	9	7	4	0
and	TE-derived	0	6	0	0	0	0
1 chromosome	mis-annotated	0	4	5	0	0	0
several similar hits	pre-miRNA	32	81	53	11	24	35
and	TE-derived	3	45	60	3	2	11
several chromosomes	mis-annotated	0	130	28	1	2	2

Number of pre-miRNAs, TE-derived pre-miRNAs and mis-annotated pre-miRNAs in function of the number of similar hits and chromosomes, in frog, human, mouse, nematode, rat, and sea squirt genomes.

In human and mouse genomes, we observed that 87.66% and 88.56% of pre-miRNAs can be mapped to a single chromosomal locus. Most of the pre-miRNAs (56 of 81 human pre-miRNAs and 38 of 53 mouse pre-miRNAs) that map to more than one chromosomal loci have only two similar hits on two chromosomes. For example, the pre-miRNA HSA-LET-7B has two hits on two chromosomes. An interesting observation is that 66.45% and 45.45% of TE-derived pre-miRNAs in human and mouse are characterized by a single similar hit.

In the case of frog, nematode, rat and sea squirt species, we observed that some pre-miRNAs that are not identified as corresponding to TEs but having many hits in several chromosomes have in fact only two occurrences on two chromosomes (data not shown). It is for example the case of 29 frog pre-miRNAs among the 32 corresponding to TEs.

The majority of precursors identified as mis-annotated pre-miRNAs because their sequence is almost entirely of TE origin have several hits on several chromosomes (Figure 5 and Table 2). In conclusion, we provide the evidences that *bona fide* pre-miRNA sequences are typically unique and encoded by a single chromosomal locus. Moreover, we show that interspersion and repetition are the most useful criterias to annotate efficiently ncRNA sequences with respect to their relationship to TEs.

### Distribution of TE families identified by ncRNAclassifier

TE-derived pre-miRNAs identified in the literature correspond often to MITEs (non-autonomous TEs of Class II) [17,20,21] and Alu (non-autonomous TEs of class I) [23]. These two types of non-autonomous TEs are well known for their stable secondary structure during transposition [5,9-11]. Therefore, we surveyed the occurences of sequences derived from MITEs, SINEs and other types of TEs when using ncRNAclassifier on the miRBase set of pre-miRNA sequences (see Table 3).

**Table 3:** TEs families involved in pre-miRNAs

		**MITE**	**Other**	**DNA**	**LTR /**	**CR1 /**	**L1**	**SINE**	**Other**	**Others**
		**mariner**	**MITEs**	**transposons**	**ERV**	**RTE**			**Non-LTR**	
Frog	TE-derived						1			
	mis-annotated									
Human	TE-derived	11	11	8	20	22	18	40	3	5
	mis-annotated	65	32		33	19	41	58		5
Mouse	TE-derived	1	13	24	5	19	1	32	3	1
	mis-annotated		5	2	17	7	11	28		7
Nematode	TE-derived		1	1	1					1
	mis-annotated		1							1
Rat	TE-derived	1	2	1		10		7		
	mis-annotated		3		1	15		11		
Sea squirt	TE-derived	1	1	1		1			2	
	mis-annotated	1	1							

TEs families involved in pre-miRNAs. The table gives the number of times TE families are identified by ncRNAclassifier in frog, human, mouse, nematode, rat and sea squirt pre-miRNAs. If a precursor contains two or more TE fragments, we counted each fragment.

A first observation is that not all TE families appear in pre-miRNA sequences, for instance we did not find Helitron, nor MuDR sequences and found only one Copia sequence in miRBase pre-miRNAs. We found that TE-derived pre-miRNAs derive mainly from non-autonomous TE sequences (66.29%). Almost all TE sequences with a stable secondary structure and a small sequence are present.

We remarked that 38.24% (97 of 253) of human mis-annotated pre-miRNAs are MITEs. Most of them (65 pre-miRNAs) are Mariner MITE (MADE1) while they represent only 0.1 to 1% of the human genome [6]. This could be because MADE1 takes an hairpin structure similar to pre-miRNAs [9,20]. As mentioned in the literature, Alu super-family and L1 super-family TEs are also present in human miRNA genes [22]. 58 mis-annotated human pre-miRNAs and 40 human TE-derived pre-miRNAs are associated to SINEs. 41 mis-annotated and 18 TE-derived pre-miRNAs are associated to L1 TEs.

In mouse, 6.49% of mis-annotated pre-miRNAs (5 of 77) and 14.14% of TE-derived pre-miRNAs (14 of 99) are MITE transposons. There are also 36.36% of mis-annotated (28 of 77) and 32.32% of TE-derived pre-miRNAs (32 of 99) that are SINE elements.

In rat, non-autonomous TEs (class I and II) correspond to 29 of 30 mis-annotated pre-miRNAs and 20 of 21 TE-derived pre-miRNAs.

In other genomes, mis-annotated pre-miRNAs and TE-derived pre-miRNAs are related to longer non-autonomous TEs than MITEs or SINEs. This is likely due to a lower quality of repetitive sequence annotations in these genomes. This size difference between long non-autonomous TEs and pre-miRNAs can explain why there are few mis-annotated pre-miRNAs in these genomes.

### Repeated and interspersed pre-miRNA sequences unrelated to TEs

Several pre-miRNA sequences were characterized by more than 20 hits interspersed on at least six chromosomes but were not identified as being related to known TEs. In order to determine why miRNA precursors having a high probability to be categorized as corresponding to TEs were not identified by ncRNAclassifier as TEs or TE-derived, we analysed a subset of these miRNAs (given in Table 4). We observed five categories: 

• The primary sequences of pre-miRNAs HSA-MIR-466, HSA-MIR-1233-1, HSA-MIR-3669, MMU-MIR-297A-6 and MMU-MIR-467g are similar to microsatellites (microsatellites are similar to tandem repeats of short sequence motifs (less than 10 nt)) [39]): HSA-MIR-466 contains microsatellites GU_*n *_and AC_*n*_; HSA-MIR-1233-1 contains microsatellites AGGGC_*n*_; and MMU-MIR-467g is composed by microsatellite AU_*n*_. The presence of microsatellites in pre-miRNA sequences explains the high number of occurrences found by BLAT, since microsatellites are very abundant in vertebrate genomes [39].

• The primary sequence of pre-miRNAs CEL-MIR-1833, CIN-MIR-4105 and XTR-MIR-427-1 are included in larger repeated sequences. The consensus reached by ncRNAclassifier is much larger than the sequence of the pre-miRNA. For example, the precursor of CIN-MIR-4105 could be extended up to 1,100 nt. We assume these sequences belong to a larger biologic entity, that is neither a known TE, a satellite nor a segmental duplication.

• The pre-miRNA sequences of CEL-MIR-1832 and CIN-MIR-4154 could not be extended in 5’ and 3’ and do not contain microsatellites. They are not related to any recognizable TE. However, further studies are necessary to confirm these annotations in their respective databases.

• Some pre-miRNAs contain TE sequences that are not recognizable by CENSOR. For example, the pre-miRNA MMU-MIR-297A-6 seems to be composed by the microsatellite CA (microsatellites annotated in Genome Browser) and is very similar to the pre-miRNA MMU-MIR-297A-5 (similarity higher than 80%, data not shown). The pre-miRNA MMU-MIR-297A-5 contains the TE ID_B1 with a low similarity score (69.33%) but ID_B1 sequence is not recognizable in the pre-miRNA MMU-MIR-297A-6. This difference of identification comes from the different mutations between MMU-MIR-297A-5 and MMU-MIR-297A-6. This TE contains a microsatellite [13] and only this microsatellite is recognizable.

• Some pre-miRNAs are linked to a TE sequence adjacent to their sequence. For example, the transposable elements ID_B1 and CR1-8_HM are respectively present in the left extended sequence of the pre-miRNA RNO-MIR-466B-2 and HSA-MIR-320D-2. It is possible that the TE “capture” the left or right adjacent sequence as the Helitron transposon captures a genomic sequence [40]. This mechanism could explain why a pre-miRNA has many similar hits in the genome.

**Table 4:** Examples of pre-miRNAs with multiple interspersed hits but not classified as TE or TE-derived by ncRNAclassifier

**pre-miRNA name**	**Genome**	**Similar hits**	**Chromosomes**
CEL-MIR-1832	Nematode	32	5
CEL-MIR-1833	Nematode	11	4
CIN-MIR-4105	Sea squirt	13	11
CIN-MIR-4154	Sea squirt	34	28
HSA-MIR-320d-2	Human	9	7
HSA-MIR-466	Human	15	12
HSA-MIR-1233-1	Human	24	6
HSA-MIR-3669	Human	9	6
MMU-MIR-297a-6	Mouse	28	10
MMU-MIR-467g	Mouse	23	12
RNO-MIR-466b-2	Rat	5	4
XTR-MIR-427-1	Frog	7	7

### Discovery of TE-derived and mis-annotated pre-miRNAs from the literature

Some studies have reported the identification of TE-derived pre-miRNAs [17,18,21]. Jordan *et al.* showed that six human pre-miRNAs (HSA-MIR-548) correspond to TEs [20]. They were called “TE-derived miRNAs”. The database microTranspoGene lists “TE-derived” pre-miRNAs of miRBase [41]. However, this database is based on release 10.0 of miRBase (the current release is 17) and there is no novel TE-derived miRNAs since 2007.

We identified with ncRNAclassifier respectively 138, 99, 4, 21 and 14 TE-derived pre-miRNAs (with TE sequences) in human, mouse, nematode, rat and sea squirt species, including 108, 88, 3, 21 and 13 not identified in the literature. We also identified 1, 235, 68, 2, 28, and 2 mis-annotated pre-miRNAs in frog, human, mouse, nematode, rat, and sea squirt which 1, 194, 57, 2, 28 and 2 were not previously identified in the literature. The six human pre-miRNAs identified by Jordan *et al.* as TE-derived have all been identified by ncRNAclassifier as mis-annotated TEs.

Our automatic method reproduced the results obtained in [17,18,20-22]. ncRNAclassifier identified most “TE-derived miRNAs” described in these studies, as well as the ones listed in microTranspoGene database.

Some have not been identified by ncRNAclassifier, for example HSA-MIR-93 and HSA-MIR-302a, which were identified in [22]. These two miRNAs contain Alu sequences of only 10 nt (the percentage of similarity was not specified in [22]). We think that CENSOR could not identify the Alu sequences because of their small size.

### Discovery of TE-derived and mis-annotated pre-miRNAs from genome annotations

It is possible to identify directly at the UCSC Genome Browser some TE-derived pre-miRNAs and mis-annotated pre-miRNAs when using their genomic coordinates. For example, the mis-annotated pre-miRNA HSA-MIR-1268 corresponds to ALU sequence in human annotation of Genome Browser.

However, some pre-miRNAs lack genomic coordinate in miRBase. For these ncRNAs, the genome annotation becomes useless, while our method is still effective. For example, the rat pre-miRNA RNO-MIR-327 lacks coordinates and BLAT finds only a portion of the sequence in the genome (less than 30%), but our method found that RNO-MIR-327 is a mis-annotated TE (95% of the sequence is RodERV21 TE sequence).

Moreover, the RepeatMasker annotations at the UCSC Genome Browser can miss some TEs. For example, the pre-miRNAs HSA-MIR-4281, MMU-MIR-680-2 and MMU-MIR-763 miRBase coordinates do not correspond to TE sequences while ncRNAclassifier found they correspond to TE-derived pre-miRNA or mis-annotated pre-miRNA: HSA-MIR-4281 is a TE-derived pre-miRNA where 57% of its sequence is a MER34_int TE; MMU-MIR-680-2 is a mis-annotated pre-miRNA where all its sequence is ERVB4_1B-LTR_MM TE sequence and MMU-MIR-763 is also a mis-annotated pre-miRNA and contains solely the Eulor5A TE sequence.

These examples show that relying on a genome annotation is not sufficient to identify mis-annotated and TE-derived pre-miRNAs. Finally, we counted respectively 4, 6, 2, 1 and 2 new human, mouse, rat, frog and sea squirt mis-annotated pre-miRNAs that have not been annotated in Genome Browser. We also counted respectively 25, 54, 4, 5 and 3 new human, mouse, rat, sea squirt and nematode TE-derived pre-miRNAs that contain TE sequence and have not been annotated. Our method confirms the genome annotations but identifies also ncRNAs without annotation.

## Conclusions

We developed an automatic method called ncRNAclassifier to classify precursor ncRNA sequences according to their similarity with TE sequences. Our method is based on the observation that a pre-ncRNA that has several occurrences widespread in the genome has a high probability to be either derived from a TE or to be mis-annotated as being a pre-ncRNA while it is a TE. The first step of ncRNAclassifier is to calculate the number of occurrences of the candidate, the number of chromosomes where appear the different occurrences and the distance between the occurrences. The second step then calculates a consensus sequence from the ten most similar occurrences to the ncRNA sequence. Finally, the last step checks if the consensus sequence corresponds to a TE in RepBase database.

Among the pre-miRNAs of miRBase, we identified hundreds of mis-annotation cases where TEs are mistaken for pre-miRNAs: 235 cases concerning the human genome and 68 for the mouse genome, with respectively 194 and 57 cases that are not mentioned in the litterature.

Recently, the validity of a set of plant miRNAs described in miRBase was re-examined [42]. The authors found that a large portion (from 6 to 100%) of plant miRNA precursors described in miRBase do not possess a canonical structure and that between 0 and 13% of plant stem-loop sequences could not be linked to canonical small RNAs identified by high-throughput sequencing. Thus, both the work of Meng *et al.*[42] and ours raise the need for improving miRNA annotations in the miRBase registry.

We plan to add features to future versions of ncRNAclassifier. One of them would be to choose the tools for identifying TE-derived ncRNAs. For example, RepeatMasker and CENSOR do not give always the same result and it is possible that CENSOR does not recognize a TE sequence in few cases while RepeatMasker can do it. We also plan to study plant pre-miRNAs databases that are known to contain pre-miRNAs that could correspond to TEs. Because the UCSC Genome Browser does not contain plant genomes, we should adapt the first step of the algorithm for other Genome Browsers such as EBI or NCBI.

Thanks to ncRNAclassifier, anyone can check very quickly if a given ncRNA hairpin sequence corresponds to a TE sequence. It requires between 30 seconds to 1 minute to treat one sequence, depending of the number of occurrences in UCSC and on the access to RepBase at EBI. ncRNAclassifier is available at the Web site: http://EvryRNA.ibisc.univ-evry.fr/.

## Methods

### ncRNAclassifier analysis of genomes

We analysed the frog, human, mouse, nematode, rat and sea squirt genomes using the command line version of ncRNAclassifier. We used the sequence and annotations present at the Genome Browser [35]: frog genome version JGI 4.1, human genome version GRG 37, mouse genome version NCBI 37, nematode genome version WS 190, rat genome version Baylor 3.4 and sea squirt genome version JGI 2.1.

## Competing interests

The authors declare that they have no competing interests.

## Authors’ contributions

ST and FT conceived the project. ST developed the software tool and performed all analyses under supervision of FT. NP supervised the biological results. ST, NP and FT wrote the manuscript. All authors read and approved the final manuscript for publication.

## Supplementary Material

Additional file 1 **Screenshot of dual annotation in Genome Broswer.** HSA-MIR-1255a is a microRNA gene present at the position 102251459 to 102251571 on chromosome 4 [24]. This locus corresponds also to the transposable element Tigger1. Click here for file

Additional file 2 **Table of miRBase pre-miRNAs from six genomes.** Analysis results of frog, human, mouse, nematode, sea squirt and rat pre-miRNAs from miRBase v.17. Click here for file
