# Supplementary figures and images for: ncRNAclassifier: a tool for detection and classification of transposable element sequences in RNA hairpins
Source: BMC Bioinformatics. 2012 Sep 25;13:246. doi: 10.1186/1471-2105-13-246 (PMC3495686; doi:10.1186/1471-2105-13-246)

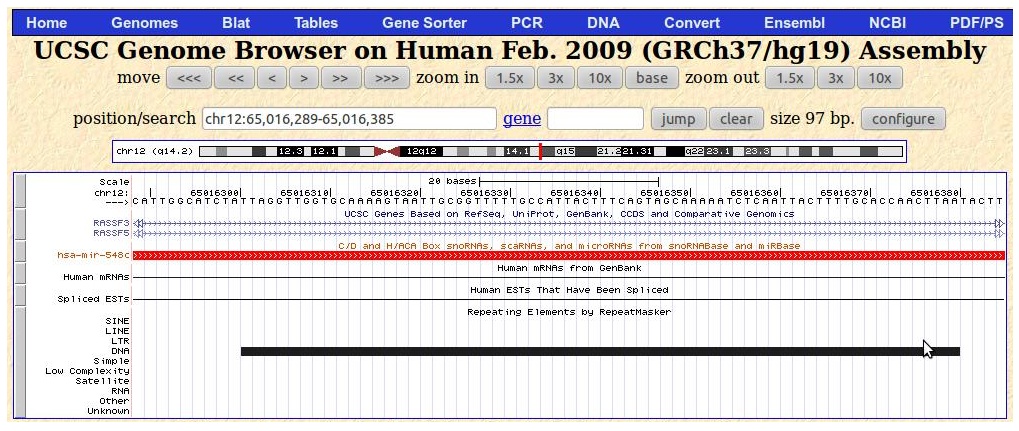

Supplement: Additional file 1 — Screenshot of dual annotation in Genome Broswer. HSA-MIR-1255a is a microRNA gene present at the position 102251459 to 102251571 on chromosome 4 [24]. This locus corresponds also to the transposable element Tigger1. [file 1471-2105-13-246-S1.png]
